# Statin adherence is lower in primary than secondary prevention: A national follow-up study of new users

**DOI:** 10.1371/journal.pone.0242424

**Published:** 2020-11-19

**Authors:** Finn Sigglekow, Simon Horsburgh, Lianne Parkin

**Affiliations:** 1 Department of Preventive and Social Medicine, Otago Medical School—Dunedin Campus, University of Otago, Dunedin, New Zealand; 2 Pharmacoepidemiology Research Network, University of Otago, Dunedin, New Zealand; University of Tasmania, AUSTRALIA

## Abstract

**Background:**

Maintaining adherence to statins reduces the risk of an initial cardiovascular disease (CVD) event in high-risk individuals (primary prevention) and additional CVD events following the first event (secondary prevention). The effectiveness of statin therapy is limited by the level of adherence maintained by the patient. We undertook a nationwide study to compare adherence and discontinuation in primary and secondary prevention patients.

**Methods:**

Dispensing data from New Zealand community pharmacies were used to identify patients who received their first statin dispensing between 2006 and 2011. The Medication Possession Ratio (MPR) and proportion who discontinued statin medication was calculated for the year following first statin dispensing for patients with a minimum of two dispensings. Adherence was defined as an MPR ≥ 0.8. Previous CVD was identified using hospital discharge records. Multivariable logistic regression was used to control for demographic and statin characteristics.

**Results:**

Between 2006 and 2011 289,666 new statin users were identified with 238,855 (82.5%) receiving the statin for primary prevention compared to 50,811 (17.5%) who received it for secondary prevention. The secondary prevention group was 1.55 (95% CI 1.51–1.59) times as likely to be adherent and 0.67 (95% CI 0.65–0.69) times as likely to discontinue statin treatment than the primary prevention group. An early gap in statin coverage increased the odds of discontinuing statin treatment.

**Conclusion:**

Adherence to statin medication is higher in secondary prevention than primary prevention. Within each group, a range of demographic and treatment factors further influences adherence.

## Introduction

Cardiovascular disease (CVD) is one of the leading causes of morbidity and mortality in New Zealand [1]. HMG-CoA reductase inhibitors (statins) are effective at reducing lipid levels and preventing CVD [2–5]. Successive versions of New Zealand primary care guidelines have recommended statins for primary prevention in patients with no history of CVD disease if their 5-year CVD risk is above 15% using risk prediction equations based on the Framingham Heart Study [6], and for secondary prevention following angina, myocardial infarction, ischaemic stroke, or transient ischaemic attack [7–10]. Statins have become a mainstay of the pharmacological prevention of CVD in New Zealand; in 2015 514,277 people (14.6% of the New Zealand population aged 18 or over) were dispensed a statin and this number has continued to rise [11, 12].

The effectiveness of statin therapy is limited by a patient’s level of adherence (the degree to which a patient conforms to a prescribed course of medication [13]). Research has demonstrated greater lipid control with higher statin adherence levels [14]. A protective relationship between statin adherence and CVD outcomes has also been found in both primary and secondary prevention populations in a number of observational studies [15–19]. In addition to the health outcome benefits accruing from the effectiveness of statin therapy, high levels of adherence lead to reduced future healthcare costs through decreased hospitalisations [20–22]. Collectively, improving statin adherence leads to better patient and health system outcomes.

The research undertaken in New Zealand to date has focused on statin adherence following an acute coronary event (i.e. secondary prevention) [23–25], and no studies internationally have compared adherence in primary and secondary prevention using the full population of statin users within a country. We undertook a large nationwide study of new users of statins to (i) compare adherence and discontinuation among patients prescribed statins for primary versus secondary prevention, (ii) explore adherence and discontinuation within primary and secondary prevention groups according to patient characteristics, and (iii) ascertain, within primary and secondary prevention groups, whether a delay in obtaining a second supply of a statin during the first year of statin therapy is associated with discontinuation.

## Materials and methods

### Data sources

The study used national demographic, pharmaceutical dispensing, and hospital discharge data supplied by the New Zealand Ministry of Health. Statin dispensing data came from the Pharmaceutical Collection database (PHARMS) which contains records of all claims by community-based pharmacists for the dispensing of prescription medications which are publicly funded [26]. Five statins were approved for use in New Zealand during the study period: atorvastatin, pravastatin, rosuvastatin, simvastatin, and a combination simvastatin and ezetimibe product. Rosuvastatin was not publicly funded so was not included in the PHARMS dispensing data. In 2010 the funding criteria for atorvastatin changed allowing all patients access, which resulted in an increase in the proportion of atorvastatin dispensed over the following years and a drop in the proportion of simvastatin dispensed. Demographic data were taken from the first statin dispensing record in PHARMS.

Hospital discharge records dating back to 1988 were obtained from the National Minimum Dataset (NMDS) [27]. Each record contains details of the principal and any additional diagnoses, as well as any procedures performed during the hospital stay. Diagnoses are coded to successive editions of the International Statistical Classification of Disease and Related Health Problems, Australian Modification (ICD9-AM and ICD10-AM during the study period) and procedures are coded to the Australian Classification of Health Interventions (ACHI) [28].

The scope of medical practice of statin prescribers, such as General Practice or Internal Medicine, was obtained from the Medical Council of New Zealand (MCNZ) who administer medical practitioners’ registrations.

### Identification of the study cohort and primary and secondary prevention groups

**Fig 1:**
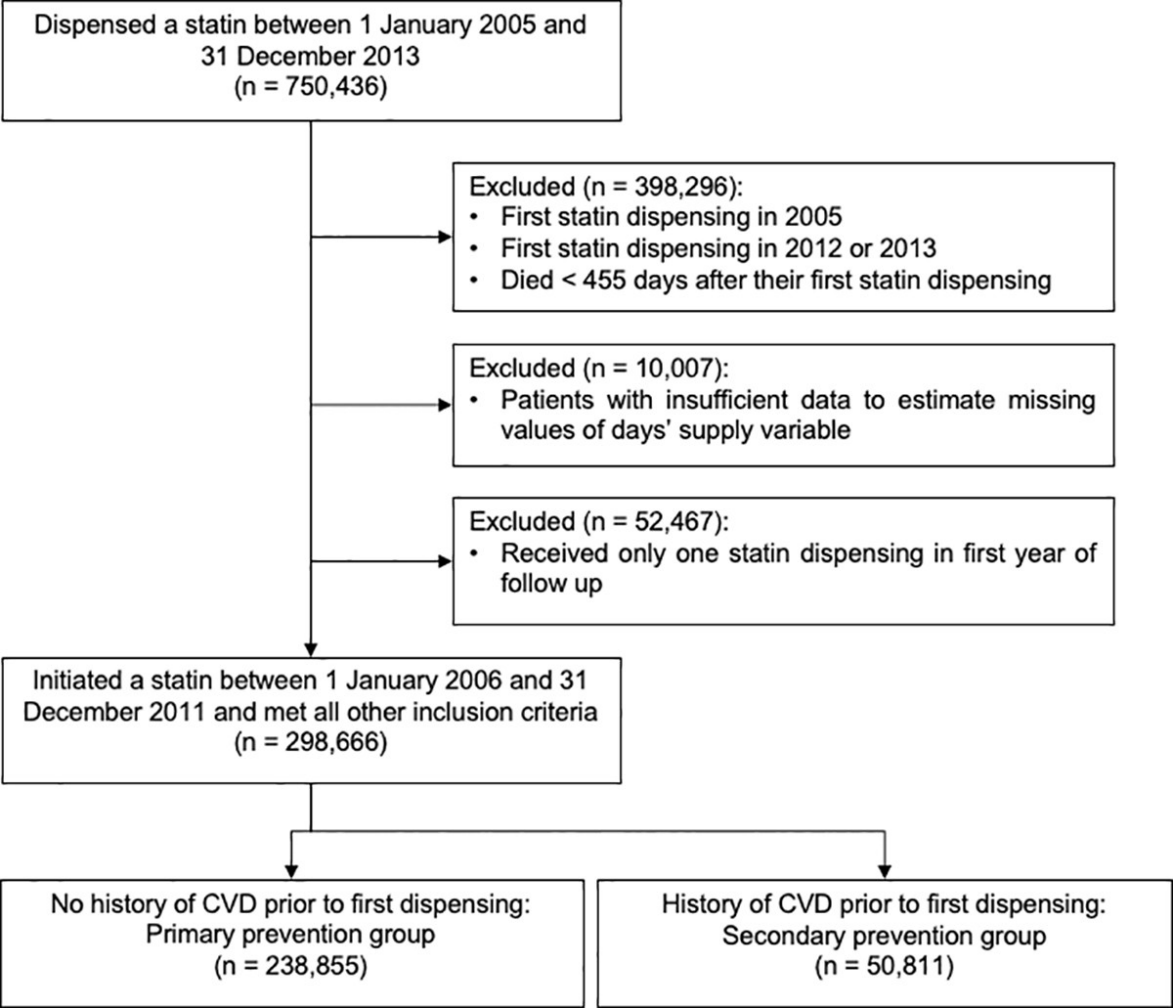
Identification of the study cohort.

The Ministry of Health identified all patients who were dispensed a publicly funded statin between 1 January 2005 and 31 December 2013, and for each patient provided us with anonymised demographic, pharmaceutical, and hospital discharge data. All patient-level data were linked with an encrypted National Health Index, a unique patient identifier used throughout the healthcare system. We excluded patients who received a statin dispensing in 2005 to ensure study members were initiating use for the first time, or following a break of at least one year (Fig 1). Patients who received their first statin dispensing after 31 December 2011 and patients who died within 455 days after their first dispensing were also excluded to ensure there was sufficient follow-up to measure discontinuation. About 15% of patients had at least one statin dispensing record in which the intended duration of the medication supplied (days’ supply) was not recorded. For the majority (about 80%) of these patients it was possible to estimate the missing value using information from the patient’s other statin dispensing records; the remaining patients were excluded. Patients with only one statin dispensing in the first year of follow-up were also excluded.

We classified patients as belonging to the secondary prevention group if they had a record in the NMDS, prior to the date of their first statin dispensing, of (i) a diagnosis of ischaemic heart disease, ischaemic stroke and/or transient ischaemic attack, and/or (ii) a clinical procedure involving angioplasty of coronary arteries, a stent inserted into the coronary arteries and/or a coronary artery bypass. A full list of diagnosis and procedure codes can be found in S1 and S2 Tables. The remaining patients were classified as belonging to the primary prevention group. For both groups, the date of the first statin dispensing was taken as the patient’s cohort entry date. Patients in the primary prevention group who had a CVD event in the follow-up period were censored.

### Statin exposure

The coverage period for each dispensing was calculated by adding the recorded days’ supply to the dispensing date. If a new dispensing occurred during the coverage period of the previous dispensing, we assumed the new supply began at the end of the coverage period. The daily dose of the first statin dispensed was calculated by multiplying the strength of the tablet dispensed by the number of tablets prescribed per day. Because statins are prescribed at different strengths depending on the type of statin [8], the daily dose was divided by the 2018 Defined Daily Dose (DDD) for the type of first statin dispensed (30 mg for simvastatin and pravastatin, 20 mg for atorvastatin) to calculate a DDD ratio. The DDD is an assumed average maintenance dose per day for a medication used for its main indication in adults [29].

### Other key variables

Other key variables included age at first statin dispensing, gender, prioritised ethnicity (using the Statistics New Zealand prioritised order of Māori; Pacific Peoples; Asian; Middle Eastern, Latin American, and African [MELAA]; Other; and European [30]) and NZDep06 [31] (an area-based index of socioeconomic deprivation). The scope of practice for each medical practitioner at the time of the first statin dispensing was identified using data from the MCNZ (S3 Table). If a medical practitioner had multiple scopes of practice, the following hierarchy was used to assign a prioritised scope: Urgent Care (vocational or provisional vocational), Internal Medicine (vocational or provisional vocational), General Practice (vocational or provisional vocational), Other vocation or provisional vocation, General Scope, and Provisional General Scope.

A modified Charlson Comorbidity Index (CCI) [32, 33] that did not include myocardial infarction, peripheral vascular disease, cerebrovascular disease, peptic ulcer disease, or diabetes without chronic complications was calculated at cohort entry using hospital discharge records from the past 5 years.

### Statistical methods

Descriptive statistics were calculated for both the primary and secondary prevention groups. Adherence and discontinuation were examined in the first year of follow-up. The Medication Possession Ratio (MPR) was calculated by dividing the total days of statin coverage in the first year by 365 days. In cases where the supply from a dispensing extended beyond the 365-day mark, the excess supply was truncated. A patient with an MPR ≥ 0.8 was classified as adherent [34]. Discontinuation was defined as a gap of > 90 days between the end of a coverage period and the next dispensing date or the end of the study period, whichever occurred first. Ninety days was chosen as this is the maximum number of days which can be supplied on a prescription in New Zealand. Patients were classified as having discontinued in the first year of follow-up if the start date of the first discontinuation period occurred < 365 days after cohort entry.

A lapse in coverage was defined as a gap ≤ 90 days between the end of the first dispensed supply and the start of the second. Any patients with a period of discontinuation before a lapse of coverage in the first year of follow-up were not eligible for inclusion in the analyses that sought to ascertain whether a lapse of coverage was associated with subsequent discontinuation.

Multivariable logistic regression was used to estimate odds ratios (ORs) and confidence intervals (95% CI) in analyses comparing adherence and discontinuation in the primary versus secondary prevention groups. ORs were adjusted for gender, age at first dispensing, prioritised ethnicity, NZDep06, modified CCI score, year of first statin dispensing, scope of practice of first statin prescriber, first statin dispensed, DDD, and days’ supply of first statin dispensing.

### Ethical approval

The study was approved by the University of Otago Human Ethics Committee (Health) (reference: HD15/031). The study was based on routinely collected anonymised data and no patient consent was required.

## Results

Overall, 289,666 new statin users between 2006 and 2011 with a minimum of two dispensings in the first year were identified, of whom 50,811 (17.5%) were hospitalised for CVD prior to cohort entry (the secondary prevention group) (Fig 1). The remaining 238,855 (82.5%) patients had no history of CVD and were assumed to have been dispensed a statin for primary prevention of CVD.

The baseline characteristics of the primary and secondary prevention groups are presented in Table 1. Compared with the primary prevention group, the secondary prevention group was older, had higher proportions of males and patients identifying as European, and higher CCI scores. However, the groups did not differ by socioeconomic status. The secondary prevention group had a higher initial dose of statins and a shorter days’ supply but there was no difference between groups in the type of statin first dispensed. The prescriber of the first statin dispensed was more likely to have a General Practice vocational registration in the primary prevention group, compared to the secondary prevention group where doctors with a General Scope registration were the top prescribers. The number of patients initiating a statin for secondary prevention dropped between 2006 and 2011, while the number dispensed statins for primary prevention fluctuated during the same period.

**Table 1:** Baseline characteristics of primary and secondary prevention groups as measured at first statin dispensing. Values are numbers (percentages) unless stated otherwise.

Characteristic	Prevention group	
	Primary (n = 238,855)	Secondary (n = 50,811)
**Gender**		
Male	128,088 (53.6)	30,705 (60.4)
Female	110,740 (46.4)	20,105 (39.6)
Unspecified	27 (0.0)	1 (0.0)
**Age at first dispensing (years)**		
< 35	6,165 (2.6)	251 (0.5)
35–44	24,009 (10.1)	2,275 (4.5)
45–54	58,069 (24.3)	7,470 (14.7)
55–64	75,062 (31.4)	12,228 (24.1)
65–74	51,705 (21.6)	12,810 (25.2)
≥ 75	23,845 (10.0)	15, 777 (31.1)
Median (IQR)	59 (50–67)	67 (57–77)
Ethnicity, prioritised*		
European	143,107 (59.9)	38,646 (76.1)
Māori	20,367 (8.5)	4,762 (9.4)
Pacific Peoples	14,461 (6.1)	1,968 (3.9)
Asian	19,840 (8.3)	1,823 (3.6)
MELAA^†^	16,291 (6.8)	1,611 (3.2)
Other	99 (0.0)	14 (0.0)
Unknown	24,690 (10.3)	1,987 (3.9)
**NZDep06 quintile**		
1 (least deprived)	33,359 (14.0)	6,407 (12.6)
2	35,360 (14.8)	6,931 (13.6)
3	44,643 (18.7)	9,624 (18.9)
4	50,701 (21.2)	11,680 (23.0)
5 (most deprived)	59,215 (24.8)	12,719 (25.0)
Unknown	15,577 (6.5)	3,450 (6.8)
**Modified CCI score**^‡^		
0	219,976 (92.1)	33,775 (66.5)
1	7,294 (3.1)	3,932 (7.7)
2	8,284 (3.5)	8,434 (16.6)
3	1,542 (0.6)	2,276 (4.5)
≥ 4	1,759 (0.7)	2,394 (4.7)
**Year of first statin dispensing**		
2006	40,021 (16.8)	11,960 (23.5)
2007	36,578 (15.3)	9,623 (18.9)
2008	42,284 (17.7)	8,358 (16.4)
2009	45,969 (19.2)	7,828 (15.4)
2010	39,262 (16.4)	6,714 (13.2)
2011	34,741 (14.5)	6,328 (12.5)
**Scope of practice of first statin prescriber**		
Vocational: General Practice	141,186 (59.1)	13,045 (25.7)
Provisional General Scope	13,502 (5.7)	15,187 (29.9)
General Scope	67,130 (28.1)	17,864 (35.2)
Vocational: Internal Medicine^§^	9,142 (3.8)	2,058 (4.1)
Vocational: Urgent Care	3,516 (1.5)	277 (0.5)
Vocational: Other	1,359 (0.6)	318 (0.6)
Unknown	2,826 (1.2)	2,022 (4.0)
Non-doctor^‖^	194 (0.1)	40 (0.1)
**First statin dispensed and daily dose (mg/day)** **		
Simvastatin^††^	211,491 (88.5)	45,006 (88.6)
< 20	34,414 (16.3)	2,567 (5.7)
20–39	124,781 (59.0)	15,207 (33.8)
40–59	50,523 (23.9)	26,346 (58.5)
60–79	318 (0.2)	133 (0.3)
≥ 80	1,455 (0.7)	753 (1.7)
DDD Ratio (Mean, SD)	0.79 (0.37)	1.08 (0.41)
Atorvastatin	27,323 (11.4)	5,795 (11.4)
< 20	11,336 (41.5)	1,120 (19.3)
20–39	11,795 (43.2)	1,559 (26.9)
40–59	3,640 (13.3)	2,179 (37.6)
60–79	44 (0.2)	42 (0.7)
≥ 80	508 (1.9)	895 (15.4)
DDD Ratio (Mean, SD)	0.98 (0.64)	1.76 (1.13)
Pravastatin	41 (0.0)	10 (0.0)
< 20	15 (36.6)	6 (60.0)
20–39	19 (46.3)	3 (30.0)
40–59	5 (12.2)	1 (10.0)
60–79	2 (4.9)	0 (0.0)
≥ 80	15 (36.6)	6 (60.0)
DDD Ratio (Mean, SD)	0.73 (0.54)	0.53 (0.32)
**Days’ supply of first statin dispensing**		
≤ 30	49,387 (20.7)	24,877 (49.0)
31–60	5,999 (2.5)	1,239 (2.4)
61–90	18,3208 (76.7)	24,651 (48.5)
≥ 91	261 (0.1)	44 (0.1)

DDD, Defined Daily Dose; SD, Standard deviation.

* In cases where patients reported multiple ethnicities, prioritised ethnicity was determined using the Statistics New Zealand prioritised order of Māori, Pacific Peoples, Asian, MELAA, Other, European [30].

^**†**^ Middle Eastern, Latin American, or African.

^**‡**^ CCI weights modified [33] removing myocardial infarction, peripheral vascular disease, cerebrovascular disease, peptic ulcer disease, and diabetes without chronic complications.

^**§**^ Includes Internal Medicine, Cardiology, Clinical Immunology, Clinical Pharmacology, Endocrinology, Gastroenterology, Geriatric Medicine, Haematology, Infectious Diseases, Medical Oncology, Nephrology, Neurology, Nuclear Medicine, Palliative Medicine, Respiratory Medicine and Rheumatology.

^**‖**^ Dentists and Registered nurses working in primary care can prescribe statins.

^**^ Denominator for overall proportions by statin type is total number of people in the relevant prevention group, whereas denominator for dosage proportion is the number of people in the relevant prevention group who were taking the statin.

^**† †**^ Includes ezetimibe with simvastatin.

Table 2 presents the mean MPR, adherence, and discontinuation in the first year of follow-up in the two groups. The mean MPR and the proportion adherent in the first year of follow-up were higher in the secondary versus the primary prevention group, while the proportion who discontinued was lower. Compared with the primary prevention group, the secondary prevention group was 1.56 (95% CI 1.52–1.60, p < 0.0001) times as likely to be adherent to statins and 0.67 (95% CI 0.65–0.69, p < 0.0001) times as likely to discontinue statin treatment.

**Table 2:** Mean MPR, adherence, and discontinuation in primary and secondary prevention groups in first year of follow-up.

Prevention group	Mean MPR	Adherent (MPR ≥ 0.8)			Discontinued		
		Adherent (%)	Unadjusted OR (95% CI)	Adjusted OR* (95% CI)	Discontinued (%)	Unadjusted OR (95% CI)	Adjusted OR* (95% CI)
Primary	0.81	62.8	1.00	1.00	29.8	1.00	1.00
Secondary	0.87	76.1	1.89 (1.84–1.93)	1.55 (1.51–1.59)	19.7	0.58 (0.57–0.59)	0.67 (0.65–0.69)

MPR, Medication Possession Ratio. OR, Odds Ratio. CI, Confidence interval.

*Adjusted for gender, age at first dispensing, prioritised ethnicity, NZDep06, modified Charlson comorbidity score, year of first statin dispensing, scope of practice of first statin prescriber, first statin dispensed, DDD ratio, and days’ supply of first statin dispensing.

Table 3 presents the mean MPR and adherence for primary and secondary prevention groups according to patient characteristics and details of the first statin dispensing. Adherence was slightly higher for females than males in the primary prevention group but lower in the secondary prevention group. Adherence in both groups increased with age and increasing comorbidity score. Compared to people of European ethnicity, adherence was lower among Māori, Pacific Peoples, and Asians in both the primary and secondary prevention groups. Patients from the lowest socioeconomic quintile had slightly lower adherence than those in the highest quintile in the primary prevention group but not in the secondary prevention group. No difference in adherence between type of statin was seen in the primary prevention group, but patients in the secondary prevention group who were first prescribed atorvastatin were slightly less adherent than those prescribed simvastatin. Adherence was lower in patients in the primary prevention group with a first dispensing lasting less than 30 days. In the primary prevention group, patients whose first statin was prescribed by a doctor with an Internal Medicine vocational registration had the highest adherence, while in the secondary prevention group adherence was higher when the statin was prescribed by a doctor with a Provisional General Scope (most practitioners in this category are junior doctors working in hospitals) or Unknown registration.

**Table 3:** Mean MPR and adherence in primary and secondary prevention groups in first year of follow-up, by patient characteristics.

Characteristic	Primary prevention group				Secondary prevention group			
	Mean	Adherent (MPR ≥ 0.80)			Mean	Adherent (MPR ≥ 0.80)		
	MPR	Adherent (%)	Unadjusted OR (95% CI)	Adjusted OR*	MPR	Adherent	Unadjusted OR (95% CI)	Adjusted OR* (95% CI)
				(95% CI)		(%)		
**Gender**								
Male	0.80	61.1	1.00	1.00	0.87	76.8	1.00	1.00
Female	0.82	64.9	1.18 (1.16–1.20)	1.10 (1.08–1.12)	0.86	75.1	0.91 (0.88–0.95)	0.90 (0.86–0.94)
Unspecified	0.86	74.1	1.82 (0.81–4.64)	2.04 (0.88–5.27)	-	-	-	-
**Age at first dispensing (years)**								
< 35	0.70	39.1	0.34 (0.32–0.36)	0.40 (0.38–0.42)	0.77	58.6	0.47 (0.37–0.61)	0.51 (0.40–0.66)
35–44	0.74	47.8	0.49 (0.47–0.50)	0.55 (0.53–0.57)	0.82	65.9	0.65 (0.59–0.71)	0.67 (0.61–0.74)
45–54	0.78	55.9	0.68 (0.66–0.69)	0.72 (0.70–0.73)	0.84	71.3	0.83 (0.78–0.89)	0.85 (0.80–0.91)
55–64	0.82	65.3	1.00	1.00	0.86	74.9	1.00	1.00
65–74	0.85	71.5	1.33 (1.30–1.37)	1.30 (1.26–1.34)	0.88	78.1	1.20 (1.13–1.27)	1.19 (1.12–1.26)
≥ 75	0.86	74.5	1.55 (1.50–1.60)	1.45 (1.40–1.50)	0.88	79.5	1.30 (1.23–1.38)	1.25 (1.18–1.33)
**Ethnicity, prioritised**								
European	0.83	67.4	1.00	1.00	0.87	78.1	1.00	1.00
Māori	0.76	51.8	0.52 (0.51–0.54)	0.63 (0.61–0.65)	0.82	66.4	0.55 (0.52–0.59)	0.63 (0.59–0.68)
Pacific Peoples	0.72	43.4	0.37 (0.36–0.38)	0.47 (0.45–0.49)	0.80	62.6	0.47 (0.43–0.52)	0.53 (0.48–0.59)
Asian	0.78	54.7	0.58 (0.57–0.60)	0.69 (0.67–0.71)	0.84	71.1	0.69 (0.62–0.77)	0.73 (0.66–0.81)
MELAA	0.82	63.0	0.83 (0.80–0.85)	0.89 (0.86–0.93)	0.87	77.2	0.95 (0.84–1.07)	0.99 (0.88–1.11)
Other	0.85	71.7	1.23 (0.80–1.93)	1.28 (0.83–2.03)	0.89	85.7	1.68 (0.46–10.81)	1.98 (0.54–12.80)
Unknown	0.82	63.6	0.85 (0.82–0.87)	0.89 (0.87–0.92)	0.88	78.1	1.00 (0.90–1.12)	1.00 (0.89–1.11)
**NZDep06 quintile**								
1 (least deprived)	0.83	65.6	1.00	1.00	0.87	77.6	1.00	1.00
2	0.82	64.9	0.97 (0.94–1.00)	1.01 (0.97–1.04)	0.87	77.7	1.01 (0.93–1.09)	1.02 (0.94–1.10)
3	0.82	65.0	0.98 (0.95–1.00)	1.01 (0.98–1.04)	0.87	77.0	0.97 (0.9–1.04)	0.99 (0.92–1.07)
4	0.82	63.9	0.93 (0.91–0.96)	1.00 (0.97–1.03)	0.87	76.7	0.95 (0.89–1.02)	1.00 (0.93–1.08)
5 (most deprived)	0.79	57.8	0.72 (0.70–0.74)	0.91 (0.88–0.94)	0.85	73.3	0.79 (0.74–0.85)	0.94 (0.87–1.01)
Unknown	0.81	61.8	0.85 (0.82–0.88)	0.95 (0.92–0.99)	0.86	76.1	0.92 (0.84–1.02)	1.01 (0.91–1.11)
**Modified Charlson comorbidity score at first statin dispensing**								
0	0.81	62.4	1.00	1.00	0.86	75.4	1.00	1.00
1	0.81	64.4	1.09 (1.04–1.15)	1.18 (1.13–1.25)	0.86	74.9	0.97 (0.90–1.05)	1.00 (0.93–1.08)
2	0.84	70.8	1.46 (1.40–1.54)	1.38 (1.32–1.46)	0.88	78.4	1.19 (1.12–1.26)	1.12 (1.11–1.25)
3	0.84	70.5	1.44 (1.29–1.61)	1.49 (1.33–1.67)	0.87	78.0	1.15 (1.04–1.28)	1.18 (1.06–1.31)
≥ 4	0.85	72.4	1.58 (1.42–1.76)	1.49 (1.34–1.66)	0.87	78.1	1.16 (1.05–1.28)	1.17 (1.05–1.29)
**Year of first statin dispensing**								
2006	0.81	62.8	1.00	1.00	0.86	74.6	1.00	1.00
2007	0.81	62.7	1.00 (0.97–1.03)	1.00 (0.97–1.03)	0.86	76.3	1.09 (1.03–1.16)	1.08 (1.01–1.15)
2008	0.81	62.9	1.00 (0.98–1.03)	1.02 (0.99–1.05)	0.86	75.9	1.07 (1.00–1.14)	1.05 (0.99–1.12)
2009	0.82	63.3	1.02 (0.99–1.05)	1.04 (1.01–1.07)	0.87	76.3	1.09 (1.02–1.17)	1.08 (1.01–1.15)
2010	0.81	62.6	0.99 (0.96–1.02)	1.02 (0.99–1.05)	0.87	77.7	1.19 (1.11–1.27)	1.17 (1.09–1.26)
2011	0.81	62.7	0.99 (0.96–1.02)	1.04 (1.00–1.07)	0.87	77.0	1.14 (1.06–1.22)	1.13 (1.04–1.22)
**Scope of practice of first statin prescriber**								
Vocational: General	0.82	64.0	1.00	1.00	0.86	73.5	1.00	1.00
Practice								
Provisional General	0.80	63.5	0.98 (0.94–1.02)	0.95 (0.91–0.98)	0.87	78.1	1.29 (1.22–1.36)	1.21 (1.14–1.28)
Scope								
General Scope	0.80	59.8	0.84 (0.82–0.86)	0.90 (0.89–0.92)	0.87	76.3	1.16 (1.10–1.22)	1.13 (1.07–1.20)
Vocational: Internal	0.85	70.9	1.37 (1.31–1.44)	1.24 (1.18–1.30)	0.87	75.9	1.14 (1.02–1.27)	1.10 (0.98–1.22)
Medicine								
Vocational: Urgent	0.76	51.8	0.61 (0.57–0.65)	0.81 (0.75–0.86)	0.83	69.7	0.83 (0.64–1.08)	0.93 (0.72–1.22)
Care								
Vocational: Other	0.79	61.2	0.89 (0.80–0.99)	1.04 (0.93–1.17)	0.85	70.8	0.87 (0.69–1.12)	0.82 (0.64–1.06)
Unknown	0.81	64.5	1.02 (0.95–1.11)	1.01 (0.93–1.09)	0.87	78.1	1.28 (1.15–1.44)	1.23 (1.10–1.38)
Non–doctor	0.80	59.3	0.82 (0.62–1.10)	0.89 (0.67–1.20)	0.88	80.0	1.44 (0.70–3.36)	1.34 (0.64–3.14)
**First statin dispensed**								
Simvastatin	0.81	62.8	1.00	1.00	0.87	76.2	1.00	1.00
Atorvastatin	0.81	63.1	1.01 (0.99–1.04)	0.98 (0.95–1.01)	0.87	75.9	0.99 (0.93–1.05)	0.86 (0.79–0.93)
Pravastatin	0.80	65.9	1.14 (0.61–2.24)	1.37 (0.72–2.73)	0.67	60.0	0.47 (0.13–1.84)	0.54 (0.15–2.13)
**DDD ratio of first statin dispensed**			0.94 (0.93–0.96)	0.98 (0.96–1.00)			1.16 (1.12–1.21)	1.23 (1.18–1.29)
**Days’ supply of first statin dispensing**								
≤ 30	0.76	59.4	0.83 (0.81–0.85)	0.80 (0.78–0.82)	0.86	76.7	1.05 (1.01–1.09)	0.94 (0.90–0.98)
31–60	0.79	62.1	0.93 (0.88–0.98)	0.85 (0.80–0.89)	0.84	70.0	0.74 (0.66–0.84)	0.70 (0.62–0.80)
61–90	0.83	63.8	1.00	1.00	0.88	75.8	1.00	1.00
≥ 91	0.92	79.7	2.23 (1.66–3.04)	2.18 (1.62–2.98)	0.92	81.8	1.43 (0.70–3.32)	1.40 (0.68–3.25)

MPR, Medication Possession Ratio. OR, Odds Ratio. CI, Confidence interval. DDD, Defined Daily Dose.

*Adjusted for gender, age at first dispensing, prioritised ethnicity, NZDep06, modified Charlson comorbidity score, year of first statin dispensing, scope of practice of first statin prescriber, first statin dispensed, DDD ratio, and days’ supply of first statin dispensing.

The analysis of discontinuation of statin therapy is presented in Table 4. Patterns of discontinuation by demographic characteristics and details of the first statin dispensing were similar to those found for adherence in Table 3.

**Table 4:** Discontinuation in primary and secondary prevention groups in first year of follow–up, by patient characteristics.

Characteristic	Primary prevention group			Secondary prevention group		
	Discontinued (%)	Unadjusted OR (95% CI)	Adjusted OR* (95% CI)	Discontinued (%)	Unadjusted OR (95% CI)	Adjusted OR* (95% CI)
**Gender**						
Male	30.6	1.00	1.00	18.4	1.00	1.00
Female	28.8	0.91 (0.90–0.93)	0.97 (0.95–0.99)	21.8	1.24 (1.19–1.30)	1.23 (1.17–1.28)
Unspecified	18.5	0.51 (0.17–1.25)	0.47 (0.16–1.17)	–	–	–
**Age at first statin dispensing (years)**						
< 35	51.7	2.86 (2.72–3.02)	2.48 (2.36–2.62)	37.8	2.46 (1.90–3.18)	2.32 (1.78–3.00)
35–44	41.5	1.90 (1.84–1.96)	1.71 (1.66–1.76)	27.0	1.49 (1.35–1.66)	1.47 (1.32–1.63)
45–54	34.7	1.42 (1.38–1.45)	1.35 (1.31–1.38)	22.6	1.18 (1.10–1.27)	1.17 (1.09–1.25)
55–64	27.2	1.00	1.00	19.8	1.00	1.00
65–74	23.4	0.81 (0.79–0.84)	0.83 (0.81–0.85)	18.0	0.89 (0.83–0.95)	0.88 (0.82–0.94)
≥ 75	22.2	0.76 (0.74–0.79)	0.80 (0.77–0.83)	18.4	0.91 (0.86–0.97)	0.90 (0.84–0.96)
Ethnicity, prioritised*						
European	26.3	1.00	1.00	18.4	1.00	1.00
Māori	38.6	1.76 (1.71–1.81)	1.48 (1.43–1.53)	27.0	1.64 (1.53–1.76)	1.45 (1.34–1.56)
Pacific Peoples	44.9	2.28 (2.20–2.36)	1.84 (1.78–1.91)	29.9	1.89 (1.71–2.09)	1.71 (1.54–1.90)
Asian	36.9	1.63 (1.58–1.69)	1.42 (1.37–1.46)	23.8	1.38 (1.24–1.54)	1.33 (1.19–1.49)
MELAA^+^	29.0	1.14 (1.10–1.18)	1.09 (1.05–1.13)	16.9	0.90 (0.79–1.03)	0.90 (0.78–1.02)
Other	23.2	0.85 (0.52–1.33)	0.80 (0.49–1.25)	7.1	0.34 (0.02–1.71)	0.29 (0.02–1.47)
Unknown	28.4	1.11 (1.08–1.15)	1.08 (1.05–1.11)	17.4	0.93 (0.83–1.05)	0.96 (0.85–1.08)
**NZ Dep06 quintile**						
1 (least deprived)	14.0	1.00	1.00	12.6	1.00	1.00
2	14.8	1.06 (1.02–1.09)	1.02 (0.99–1.06)	13.6	1.01 (0.93–1.10)	1.00 (0.92–1.09)
3	18.7	1.07 (1.04–1.10)	1.03 (1.00–1.07)	18.9	1.05 (0.97–1.14)	1.02 (0.94–1.11)
4	21.2	1.11 (1.07–1.14)	1.03 (0.99–1.06)	23.0	1.05 (0.97–1.14)	0.99 (0.92–1.08)
5 (most deprived)	24.8	1.38 (1.34–1.42)	1.11 (1.08–1.15)	25.0	1.28 (1.19–1.38)	1.11 (1.02–1.20)
Unknown	6.5	1.17 (1.12–1.22)	1.06 (1.01–1.10)	6.8	1.08 (0.97–1.20)	1.01 (0.91–1.13)
**Modified Charlson comorbidity score at first statin dispensing**						
0	30.1	1.00	1.00	20.0	1.00	1.00
1	29.4	0.97 (0.92–1.02)	0.87 (0.84–0.93)	21.5	1.09 (1.01–1.19)	1.04 (0.96–1.13)
2	24.4	0.75 (0.71–0.79)	0.77 (0.73–0.81)	18.4	0.90 (0.85–0.96)	0.88 (0.83–0.94)
3	24.2	0.74 (0.66–0.83)	0.70 (0.62–0.79)	19.3	0.96 (0.86–1.06)	0.90 (0.81–1.01)
≥ 4	24.0	0.74 (0.66–0.82)	0.76 (0.68–0.85)	18.6	0.91 (0.82–1.02)	0.87 (0.78–0.97)
**Year of first statin dispensing**						
2006	29.5	1.00	1.00	20.2	1.00	1.00
2007	29.6	1.01 (0.98–1.04)	1.00 (0.97–1.03)	19.7	0.97 (0.91–1.04)	0.98 (0.91–1.05)
2008	29.8	1.01 (0.98–1.05)	1.00 (0.97–1.03)	20.0	0.99 (0.92–1.06)	1.00 (0.93–1.07)
2009	29.3	0.99 (0.96–1.02)	0.98 (0.95–1.01)	19.7	0.97 (0.91–1.05)	0.97 (0.90–1.05)
2010	30.0	1.02 (0.99–1.05)	1.00 (0.97–1.03)	19.0	0.93 (0.86–1.00)	0.93 (0.86–1.01)
2011	30.6	1.05 (1.02–1.09)	1.02 (0.98–1.05)	19.4	0.95 (0.88–1.03)	0.98 (0.90–1.07)
**Scope of practice of first statin prescriber**					
Vocational: General	28.4	1.00	1.00	21.1	1.00	1.00
Practice						
Provisional General	31.3	1.15 (1.11–1.19)	1.18 (1.13–1.23)	18.5	0.85 (0.80–0.90)	0.91 (0.86–0.97)
Scope						
General Scope	32.6	1.22 (1.20–1.25)	1.14 (1.12–1.17)	19.7	0.91 (0.86–0.96)	0.95 (0.90–1.01)
Vocational: Internal	24.5	0.82 (0.78–0.86)	0.91 (0.87–0.96)	20.1	0.94 (0.84–1.05)	0.99 (0.88–1.12)
Medicine						
Vocational: Urgent Care	38.8	1.60 (1.49–1.71)	1.24 (1.16–1.33)	25.6	1.29 (0.97–1.68)	1.15 (0.87–1.51)
Vocational: Other	33.7	1.28 (1.15–1.44)	1.12 (1.00–1.26)	23.0	1.11 (0.85–1.44)	1.21 (0.92–1.57)
Unknown	29.1	1.04 (0.95–1.12)	1.05 (0.97–1.14)	18.7	0.86 (0.76–0.97)	0.92 (0.81–1.04)
Non–doctor	35.1	1.36 (1.01–1.82)	1.25 (0.92–1.68)	22.5	1.08 (0.49–2.18)	1.16 (0.52–2.36)
**First statin dispensed**						
Simvastatin	29.8	1.00	1.00	19.8	1.00	1.00
Atorvastatin	29.9	1.01 (0.98–1.03)	1.03 (0.99–1.06)	19.2	0.96 (0.90–1.03)	1.14 (1.05–1.24)
Pravastatin	19.5	0.57 (0.25–1.18)	0.46 (0.19–0.96)	50.0	4.05 (1.13–14.57)	3.23 (0.89–11.74)
**DDD ratio of first statin dispensed**	–	1.02 (1.00–1.04)	0.98 (0.96–1.00)	–	0.78 (0.75–0.81)	0.75 (0.70–0.78)
**Days’ supply of first statin dispensing**					
≤ 30	33.2	1.22 (1.19–1.25)	1.23 (1.20–1.26)	19.6	0.99 (0.94–1.03)	1.04 (0.99–1.09)
31–60	28.3	0.97 (0.91–1.03)	1.04 (0.98–1.10)	19.9	1.00 (0.87–1.15)	1.03 (0.89–1.19)
61–90	28.9	1.00	1.00	19.9	1.00	1.00
≥ 91	22.6	0.72 (0.53–0.95)	0.74 (0.55–0.98)	15.9	0.76 (0.31–1.61)	0.75 (0.31–1.60)

OR, Odds Ratio. CI, Confidence interval. DDD, Defined Daily Dose.

*Adjusted for gender, age at first dispensing, prioritised ethnicity, NZDep06, modified Charlson comorbidity score, year of first statin dispensing, scope of practice of first statin prescriber, first statin dispensed, DDD ratio, and days’ supply of first statin dispensing.

The results of the analyses which explored the impact of a lapse in coverage between the first and second dispensings on subsequent discontinuation are shown in Table 5. The analyses were based on 266,420 patients (after the exclusion of patients with an episode of discontinuation before a lapse of coverage in the first year of follow-up), with 217,679 (81.7%) and 48,741 (18.3%) receiving a statin for primary and secondary prevention, respectively. In total, 103,703 (47.6%) patients in the primary prevention group had a lapse in coverage (median 12 days, interquartile range 4–31 days) between the first and second dispensings, and were 1.51 (CI 95% 1.48–1.54, p < 0.0001) times as likely to discontinue than patients with no lapse. In the secondary prevention group, 13,732 (28.2%) patients had a lapse in coverage (median 8 days, interquartile range 3–25 days) and were 1.60 (CI 95% 1.52–1.69, p < 0.0001) times as likely to discontinue than patients with no lapse.

**Table 5:** Impact of a lapse of coverage between the first and second statin dispensing on odds of subsequent discontinuation of statin.

	Primary prevention group			Secondary prevention group		
	Discontinued (%)	Unadjusted OR (95% CI)	Adjusted OR* (95% CI)	Discontinued (%)	Unadjusted OR (95% CI)	Adjusted OR* (95% CI)
Lapse of coverage between first and second dispensing	27.0	1.56 (1.53–1.59)	1.51 (1.48–1.54)	21.2	1.59 (1.51–1.67)	1.60 (1.52–1.69)
No lapse of coverage	19.2	1.00	1.00	14.4	1.00	1.00

OR, Odds Ratio. CI, Confidence interval.

*Adjusted for gender, age at first dispensing, prioritised ethnicity, NZDep06, modified Charlson comorbidity score, year of first statin dispensing, scope of practice of first statin prescriber, first statin dispensed, DDD ratio, and days’ supply of first statin dispensing.

## Discussion

In this nationwide study of new users of statins, we found that patients who had been prescribed a statin for secondary prevention of CVD had higher adherence and lower discontinuation than patients prescribed statins for primary prevention. Within the primary prevention group, adherence and discontinuation levels differed by age, comorbidity level, ethnicity and initial prescriber scope of practice. In the secondary prevention group, adherence and discontinuation also varied by age, comorbidity level and ethnicity, as well as initial prescriber scope of practice. However, the type of statin initially dispensed was also associated with both adherence and discontinuation in the secondary prevention group. These patterns of associations remained unchanged when an MPR ≥ 0.90 was used as a threshold for adherence in additional sensitivity analyses (not shown). Of particular note, we found that a lapse in coverage between the first and second dispensings was a predictor of subsequent discontinuation of statin therapy.

Our finding that 76% of patients in the secondary prevention group had an MPR ≥ 0.8 in the first year of follow-up is consistent with results from previous New Zealand investigations [23–25]. A national study of patients aged 35–84 years who were discharged from hospital in 2007 following an admission for angina or acute coronary syndrome (ACS) found that 59% of patients had a statin dispensing ratio (SDR) ≥ 0.8 in the first year of follow-up [25], while a related national study confined to patients discharged following an ACS admission in the same year found that 69% of patients had an MPR ≥ 0.8 in the first year of follow-up [23]. In the third study by the same research group, 75% of patients discharged from two hospitals following ACS in the years 2007–2011 had an MPR ≥ 0.8 in the first year of follow-up [24]. These investigations differed somewhat from our study in that they were confined to patients with acute coronary events, and SDR and MPR values were calculated for all patients, including those who were not dispensed a statin in the first year of follow-up. To the best of our knowledge, this study is the first to provide national-level information about adherence among New Zealand patients dispensed statins for primary prevention, so no direct comparison with previous work is possible.

In contrast to our nationwide study, research on statin adherence and discontinuation in primary and secondary prevention internationally has focussed on specific sub-populations or samples from the general population. Nonetheless, the differences we observed between primary and secondary prevention groups is consistent with other research internationally. For instance, higher levels of discontinuation and lower adherence in primary versus secondary prevention have been reported among diverse patient groups including middle-aged members of a health insurance plan in Quebec, Canada [35], elderly patients in Ontario, Canada [36], patients over 45 in Finland [37], patients enrolled in managed care organisations in the United States [38, 39], members of a health maintenance organisation in Israel [40], and patients attending practices contributing to a general practice research database in the United Kingdom [41].

Our finding that statin adherence differed by age, gender, ethnicity, and comorbidities is consistent with findings from elsewhere [16, 34]. With the exception of gender, we found that these factors influenced statin adherence similarly in both primary and secondary prevention groups. Our observation that, after adjusting for other factors, atorvastatin was associated with slightly lower adherence than simvastatin in the secondary prevention group is consistent with the findings of a study in the United States [42]. Conversely, a study in the United Kingdom based on a secondary prevention group found no differences in adherence to atorvastatin and simvastatin [43], and Finnish research reported adherence to be higher for atorvastatin than simvastatin in both a primary prevention [44] and mixed-prevention cohort [37]. In contrast to some overseas studies [44–46], we did not find an association between socioeconomic status and statin adherence except for patients living in the most deprived areas. This may partly reflect the variation in how socioeconomic status is measured across countries and studies.

Our study had several strengths and limitations. One strength is that our study uses a cohort of all patients in New Zealand who initiated statin therapy during the study period, thereby minimising selection bias and improving statistical power. For a community pharmacist to be reimbursed for a dispensing they must submit a claim that is recorded in PHARMS. This means the statin dispensing data for funded statins are likely to be virtually complete for the whole of New Zealand during the study period. We believe rosuvastatin, a non-funded statin, is rarely prescribed and its non-inclusion will not impact our results. The use of the NMDS allowed us to identify patients with a history of being admitted to hospital for CVD as all public hospitals in New Zealand are required to report all inpatient and day patient discharges with diagnosis data.

Our study was based on dispensing information and we did not have access to prescription data, therefore patients who were prescribed a statin but never filled their prescription would not have been included in our cohort. However, the lack of prescribing data may not have been an issue for our estimates of adherence in the secondary prevention group as previous New Zealand research has found that most patients who were discharged from hospital following an acute coronary syndrome event filled their statin prescription [24]. The use of dispensing data to calculate adherence is based on an assumption that medication possession equates to consumption, however this limitation applies to nearly all methods of measuring medication adherence. Our classification of patients into primary and secondary prevention groups was based on hospital discharge records. A recent study found 39% of patients with a history of CVD hospitalisation did not have that history recorded in their primary care notes [47]. This missing information might have an impact on the type and strength of statin the medical practitioner prescribes and the type of conversation they have around the reason for taking the statin. Finally, we did not assess the impact of other factors which may influence adherence behaviour, such as depression and alcohol use [16, 48, 49], due to the absence or poor capture of information on these factors in the data available to us.

Our data do not explain why adherence was lower in the primary prevention group than in the secondary prevention group. The motivation to take a statin regularly is likely to be higher in the latter group due to their having already experienced one of the events taking a statin is intended to prevent. The absolute benefits of statin therapy also increase as cardiovascular risk increases while the risk of statin-induced side effects remains largely static regardless of cardiovascular risk, leading to a better benefit-risk profile in secondary prevention patients and greater motivation [38, 40].

There are some important implications for statin prescribers, and the wider healthcare delivery system, from our study. The most novel is that the late filling of a statin prescription is associated with subsequent discontinuation in the first year of therapy. This delay therefore provides an important indicator for clinicians to identify and intervene early in patients at increased risk of statin therapy discontinuation. Our finding of differences in the levels of adherence and risk of discontinuation across population groups based on prescription refill data speak not only to differences in adherence behaviour between these groups, but also to issues with access to medicines for these groups. While much is often discussed about how medication adherence might be improved by interventions targeted at individuals’ adherence behaviour, our results are also consistent with other research that has identified barriers in the equitable access to healthcare for Māori and Pacific populations in particular within New Zealand [50–52]. These groups are also at significantly higher risk of death from cardiac events [53], and it is incumbent on the health system to consider not just the behavioural interventions which can be used to improve adherence in these populations, but the way in which the system itself delivers healthcare [54].

In conclusion, our study showed adherence to statin medication is lower in primary prevention than secondary prevention, is associated with demographic factors, and discontinuation of medication can be predicted by late filling of the second prescription. Using this information, strategies can be developed to identify patients at increased risk of poor adherence early in their statin therapy and guide further research into how healthcare can be better delivered to increase patient adherence and reduce the risk of CVD.

## Supporting information

S1 Table ICD codes for cardiovascular disease diagnoses. (DOCX)

S2 Table ACHI codes for cardiovascular disease procedures. (DOCX)

S3 Table Medical Council of New Zealand (MCNZ) definitions. (DOCX)

## References

[1] Ministry of Health. Health loss in New Zealand 1990–2013: a report from the New Zealand Burden of Diseases, Injuries and Risk Factors Study. Wellington: Ministry of Health; 2016.

[2] Taylor F, Huffman MD, Macedo AF, Moore THM, Burke M, Davey Smith G, et al Statins for the primary prevention of cardiovascular disease. Cochrane database Syst Rev. 2013;30: CD004816 10.1002/14651858.CD004816.pub5 23440795 PMC6481400

[3] Cholesterol Treatment Trialists’ (CTT) Collaborators. Efficacy and safety of cholesterol-lowering treatment: prospective meta-analysis of data from 90,056 participants in 14 randomised trials of statins. Lancet. 2005;366: 1267–78. 10.1016/S0140-6736(05)67394-1 16214597

[4] Cholesterol Treatment Trialists’ (CTT) Collaborators. Efficacy of cholesterol-lowering therapy in 18 686 people with diabetes in 14 randomised trials of statins: a meta-analysis. Lancet. 2008;371: 117–125. 10.1016/S0140-6736(08)60104-X 18191683

[5] Cholesterol Treatment Trialists’ (CTT) Collaboration. Efficacy and safety of more intensive lowering of LDL cholesterol: a meta-analysis of data from 170,000 participants in 26 randomised trials. Lancet. 2010;376: 1670–81. 10.1016/S0140-6736(10)61350-5 21067804 PMC2988224

[6] Pylypchuk R, Wells S, Kerr A, Poppe K, Riddell T, Harwood M, et al Cardiovascular disease risk prediction equations in 400 000 primary care patients in New Zealand: a derivation and validation study. Lancet. 2018;391: 1897–1907. 10.1016/S0140-6736(18)30664-0 29735391

[7] Ministry of Health. Cardiovascular disease risk assessment and management for primary care. Wellington: Ministry of Health; 2018.

[8] New Zealand Guidelines Group. New Zealand primary care handbook 2012. 3rd ed Wellington: New Zealand Guidelines Group; 2012.

[9] New Zealand Guidelines Group. Assessment and management of cardiovascular risk. Wellington: New Zealand Guidelines Group; 2003.

[10] New Zealand Guidelines Group. New Zealand Cardiovascular Guidelines Handbook: a summary resource for primary care practitioners. 2nd ed Wellington: New Zealand Guidelines Group; 2009.

[11] Ministry of Health. Pharmaceutical data web tool version 03 August 2020 (data extracted from Pharmaceutical Collection on 05 March 2020). 2020 [cited 31 Aug 2020]. Available: https://minhealthnz.shinyapps.io/pharmaceutical_data_web_tool/

[12] StatsNZ. Infoshare Population Estimates. 2020 [cited 31 Aug 2020]. Available: http://archive.stats.govt.nz/infoshare/SelectVariables.aspx?pxID=ca07519d-687e-47b6-9e75-42e5d37a6452

[13] Vrijens B, De Geest S, Hughes DA, Przemyslaw K, Demonceau J, Ruppar T, et al A new taxonomy for describing and defining adherence to medications. Br J Clin Pharmacol. 2012;73: 691–705. 10.1111/j.1365-2125.2012.04167.x 22486599 PMC3403197

[14] Watanabe JH, Bounthavong M, Chen T. Revisiting the medication possession ratio threshold for adherence in lipid management. Curr Med Res Opin. 2013;29: 175–180. 10.1185/03007995.2013.766164 23320610

[15] Ho PM, Bryson CL, Rumsfeld JS. Medication adherence: its importance in cardiovascular outcomes. Circulation. 2009;119: 3028–3035. 10.1161/CIRCULATIONAHA.108.768986 19528344

[16] Hope HF, Binkley GM, Fenton S, Kitas GD, Verstappen SMM, Symmons DPM. Systematic review of the predictors of statin adherence for the primary prevention of cardiovascular disease. Zeeb H, editor. PLoS One. 2019;14: e0201196 10.1371/journal.pone.0201196 30653535 PMC6336256

[17] Gehi AK, Ali S, Na B, Whooley MA. Self-reported medication adherence and cardiovascular events in patients with stable coronary heart disease: the heart and soul study. Arch Intern Med. 2007;167: 1798–1803. 10.1001/archinte.167.16.1798 17846400 PMC2789555

[18] Slejko JF, Ho PM, Anderson HD, Nair K V., Sullivan PW, Campbell JD. Adherence to statins in primary prevention: yearly adherence changes and outcomes. J Manag Care Pharm. 2014;20: 51–57. 10.18553/jmcp.2014.20.1.51 24372460 PMC10438208

[19] Martin-Ruiz E, Olry-de-Labry-Lima A, Ocaña-Riola R, Epstein D. Systematic Review of the Effect of Adherence to Statin Treatment on Critical Cardiovascular Events and Mortality in Primary Prevention. J Cardiovasc Pharmacol Ther. 2018;23: 200–215. 10.1177/1074248417745357 29343082

[20] Pittman DG, Chen W, Bowlin SJ, Foody JM. Adherence to statins, subsequent healthcare costs, and cardiovascular hospitalizations. Am J Cardiol. 2011;107: 1662–1666. 10.1016/j.amjcard.2011.01.052 21439533

[21] Dragomir A, Côté R, White M, Lalonde L, Blais L, Bérard A, et al Relationship between Adherence Level to Statins, Clinical Issues and Health-Care Costs in Real-Life Clinical Setting. Value Heal. 2010;13: 87–94. 10.1111/j.1524-4733.2009.00583.x 19695008

[22] Cutler RL, Fernandez-Llimos F, Frommer M, Benrimoj C, Garcia-Cardenas V. Economic impact of medication non-adherence by disease groups: a systematic review. BMJ Open. 2018;8: e016982 10.1136/bmjopen-2017-016982 29358417 PMC5780689

[23] Grey C, Jackson R, Wells S, Thornley S, Marshall R, Crengle S, et al Maintenance of statin use over 3 years following acute coronary syndromes: a national data linkage study (ANZACS-QI-2). Heart. 2014;100: 770–774. 10.1136/heartjnl-2013-304960 24436219

[24] Kerr AJ, Turaga M, Grey C, Lee M, McLachlan A, Devlin G. Initiation and maintenance of statins and aspirin after acute coronary syndromes (ANZACS-QI 11). J Prim Health Care. 2016;8: 238–249. 10.1071/HC16013 29530207

[25] Thornley S, Marshall R, Chan WC, Kerr A, Harrison J, Jackson G, et al Four out of ten patients are not taking statins regularly during the 12 months after an acute coronary event. Eur J Prev Cardiol. 2012;19: 349–357. 10.1177/1741826711403069 21450568

[26] Ministry of Health. Pharmaceutical Collection. 2018 [cited 4 Aug 2018]. Available: https://www.health.govt.nz/nz-health-statistics/national-collections-and-surveys/collections/pharmaceutical-collection

[27] Ministry of Health. National Minimum Dataset (hospital events). 2018 [cited 4 Aug 2018]. Available: https://www.health.govt.nz/nz-health-statistics/national-collections-and-surveys/collections/national-minimum-dataset-hospital-events

[28] Ministry of Health. ICD-10-AM/ACHI/ACS. 2018 [cited 4 Aug 2018]. Available: https://www.health.govt.nz/nz-health-statistics/classification-and-terminology/icd-10-am-achi-acs

[29] WHO Collaborating Centre for Drug Statistics Methodology. Definition and general considerations. 2018 [cited 4 Aug 2018]. Available: https://www.whocc.no/ddd/definition_and_general_considera/

[30] Ministry of Health. HISO 10001:2017 ethnicity data protocols. Wellington: Ministry of Health; 2017.

[31] Salmond C, Crampton P, Atkinson J. NZDep2006 Index of Deprivation user’s manual. Wellington: University of Otago; 2007.

[32] Quan H, Sundararajan V, Halfon P, Fong A, Burnand B, Luthi JC, et al Coding algorithms for defining comorbidities in ICD-9-CM and ICD-10 administrative data. Med Care. 2005;43: 1130–1139. 10.1097/01.mlr.0000182534.19832.83 16224307

[33] Quan H, Li B, Couris CM, Fushimi K, Graham P, Hider P, et al Updating and validating the charlson comorbidity index and score for risk adjustment in hospital discharge abstracts using data from 6 countries. Am J Epidemiol. 2011;173: 676–682. 10.1093/aje/kwq433 21330339

[34] Mann DM, Woodward M, Muntner P, Falzon L, Kronish I. Predictors of nonadherence to statins: a systematic review and meta-analysis. Ann Pharmacother. 2010;44: 1410–1421. 10.1345/aph.1P150 20702755 PMC3641194

[35] Perreault S, Blais L, Lamarre D, Dragomir A, Berbiche D, Lalonde L, et al Persistence and determinants of statin therapy among middle-aged patients for primary and secondary prevention. Br J Clin Pharmacol. 2005;59: 564–573. 10.1111/j.1365-2125.2005.02355.x 15842555 PMC1884848

[36] Jackevicius CA, Mamdani M, Tu J V. Adherence with statin therapy in elderly patients with and without acute coronary syndromes. JAMA. 2002;288: 462–7. 10.1001/jama.288.4.462 12132976

[37] Aarnio EJ, Martikainen JA, Helin-Salmivaara A, Huupponen RK, Hartikainen JEK, Peura PK, et al Register-based predictors of adherence among new statin users in Finland. J Clin Lipidol. 2014;8: 117–125. 10.1016/j.jacl.2013.09.008 24528692

[38] Ellis JJ, Erickson SR, Stevenson JG, Bernstein SJ, Stiles RA, Fendrick AM. Suboptimal statin adherence and discontinuation in primary and secondary prevention populations: should we target patients with the most to gain? J Gen Intern Med. 2004;19: 638–645. 10.1111/j.1525-1497.2004.30516.x 15209602 PMC1492382

[39] Chapman RH, Benner JS, Petrilla AA, Tierce JC, Collins SR, Battleman DS, et al Predictors of adherence with antihypertensive and lipid-lowering therapy. Arch Intern Med. 2005;165: 1147–1152. 10.1001/archinte.165.10.1147 15911728

[40] Chodick G, Shalev V, Gerber Y, Heymann AD, Silber H, Simah V, et al Long-term persistence with statin treatment in a not-for-profit health maintenance organization: a population-based retrospective cohort study in Israel. Clin Ther. 2008;30: 2167–2179. 10.1016/j.clinthera.2008.11.012 19108805

[41] Vinogradova Y, Coupland C, Brindle P, Hippisley-Cox J. Discontinuation and restarting in patients on statin treatment: prospective open cohort study using a primary care database. BMJ. 2016;353: i3305 10.1136/bmj.i3305 27353261 PMC4925919

[42] Muntner P, Yun H, Sharma P, Delzel E, Kent ST, Kilgore ML, et al Ability of low antihypertensive medication adherence to predict statin discontinuation and low statin adherence in patients initiating treatment after a coronary event. Am J Cardiol. 2014;114: 826–831. 10.1016/j.amjcard.2014.06.009 25103917

[43] Carey IM, DeWilde S, Shah SM, Harris T, Whincup PH, Cook DG. Statin use after first myocardial infarction in UK men and women from 1997 to 2006: who started and who continued treatment? Nutr Metab Cardiovasc Dis. 2012;22: 400–408. 10.1016/j.numecd.2010.09.010 21194912

[44] Aarnio E, Martikainen J, Winn AN, Huupponen R, Vahtera J, Korhonen MJ. Socioeconomic inequalities in statin adherence under universal coverage: does sex matter? Circ Cardiovasc Qual Outcomes. 2016;9: 704–713. 10.1161/CIRCOUTCOMES.116.002728 27756795

[45] Xie Z, St. Clair P, Goldman DP, Joyce G. Racial and ethnic disparities in medication adherence among privately insured patients in the United States. Ruiz JM, editor. PLoS One. 2019;14: e0212117 10.1371/journal.pone.0212117 30763400 PMC6375669

[46] Erickson SR, Bravo M, Tootoo J. Geosocial Factors Associated With Adherence to Statin Medications. Ann Pharmacother. 2020;54: 1194–1202. 10.1177/1060028020934879 32522004

[47] Wells S, Poppe KK, Selak V, Kerr A, Pylypchuk R, Wu B, et al Is general practice identification of prior cardiovascular disease at the time of CVD risk assessment accurate and does it matter? N Z Med J. 2018;131: 10–20. 29771897

[48] Osterberg L, Blaschke T. Adherence to Medication. N Engl J Med. 2005;353: 487–497. 10.1056/NEJMra050100 16079372

[49] Grenard JL, Munjas BA, Adams JL, Suttorp M, Maglione M, McGlynn EA, et al Depression and medication adherence in the treatment of chronic diseases in the United States: a meta-analysis. J Gen Intern Med. 2011;26: 1175–1182. 10.1007/s11606-011-1704-y 21533823 PMC3181287

[50] Ministry of Health. Annual Data Explorer 2018/19: New Zealand Health Survey [Data File]. 2019 [cited 27 Feb 2020]. Available: https://minhealthnz.shinyapps.io/nz-health-survey-2018-19-annual-data-explorer/

[51] Jatrana S, Crampton P, Norris P. Ethnic differences in access to prescription medication because of cost in New Zealand. J Epidemiol Community Heal. 2011;65: 454–460. 10.1136/jech.2009.099101 20466707

[52] Palmer SC, Gray H, Huria T, Lacey C, Beckert L, Pitama SG. Reported Māori consumer experiences of health systems and programs in qualitative research: a systematic review with meta-synthesis. Int J Equity Health. 2019;18: 163 10.1186/s12939-019-1057-4 31660988 PMC6816189

[53] Grey C, Jackson R, Wells S, Wu B, Poppe K, Harwood M, et al Trends in ischaemic heart disease: patterns of hospitalisation and mortality rates differ by ethnicity (ANZACS-QI 21). N Z Med J. 2018;131: 21–31. 30001303

[54] Waitangi Tribunal. Hauora: report on stage one of the Health Services and Outcomes Kaupapa Inquiry WAI 2575: Waitangi Tribunal Report. Wellington, New Zealand; 2019.

