# Supplementary material for: Statin adherence is lower in primary than secondary prevention: A national follow-up study of new users
Source: PLoS One. 2020 Nov 19;15(11):e0242424. doi: 10.1371/journal.pone.0242424 (PMC7676659; doi:10.1371/journal.pone.0242424)
Supplement: S1 Table — (DOCX) [file pone.0242424.s001.docx]

**S1 Table: ICD codes for cardiovascular disease diagnoses**

| **Diagnosis** | **ICD version** | **Clinical Code** | **Clinical Description** |
| --- | --- | --- | --- |
| Ischaemic heart disease | ICD9 – AM | 410 | Acute myocardial infarction |
|  |  | 411 | Other acute and sub-acute forms of ischaemic heart disease |
|  |  | 412 | Old myocardial infarction |
|  |  | 413 | Angina pectoris |
|  |  | 414 | Other forms of chronic ischaemic heart disease |
|  | ICD10 – AM | I20 | Angina pectoris |
|  |  | I21 | Acute myocardial infarction |
|  |  | I22 | Subsequent myocardial infarction |
|  |  | I23 | Certain current complications following acute myocardial infarction |
|  |  | I24 | Other acute ischaemic heart disease |
|  |  | I25 | Chronic ischaemic heart disease |
| Ischaemic stroke | ICD9 – AM | 43301 | Basilar artery with cerebral infarction |
|  |  | 43311 | Carotid artery with cerebral infarction |
|  |  | 43321 | Vertebral artery with cerebral infarction |
|  |  | 43331 | Multiple and bilateral with cerebral infarction |
|  |  | 43381 | Other specified precerebral artery with cerebral infarction |
|  |  | 43391 | Unspecified precerebral artery with cerebral infarction |
|  |  | 43401 | Cerebral thrombosis with cerebral infarction |
|  |  | 43411 | Cerebral embolism with cerebral infarction |
|  |  | 43491 | Cerebral artery occlusion, unspecified with cerebral infarction |
|  | ICD10 – AM | I63 | Cerebral infarction |
| Transient ischaemic attack | ICD9 – AM | 435 | Transient ischaemic attack |
|  | ICD10 – AM | G45 | Transient cerebral ischaemic attacks and related syndromes |
