# Supplementary material for: Statin adherence is lower in primary than secondary prevention: A national follow-up study of new users
Source: PLoS One. 2020 Nov 19;15(11):e0242424. doi: 10.1371/journal.pone.0242424 (PMC7676659; doi:10.1371/journal.pone.0242424)
Supplement: S2 Table — (DOCX) [file pone.0242424.s002.docx]

**S2 Table: ACHI codes for cardiovascular disease procedures**

|  | **Clinical Code** | **Clinical description** |
| --- | --- | --- |
| **ACHI edition** | |  |
| 1^st^, 2^nd^, 3^rd^ | 35304-00, 35304-01 | Percutaneous or open transluminal balloon angioplasty of 1 coronary artery |
|  | 35305-00, 35305-01 | Percutaneous or open transluminal balloon angioplasty of 2 or more coronary arteries |
|  | 35310-00 to 35310-05 | Percutaneous or open insertion of >=1 transluminal stent into single/multiple coronary artery |
| 1^st^, 2^nd^, 3^rd^, 6^th^, 8^th^ | 38497-00 to 38497-07 | Coronary artery bypass, using >=1 saphenous vein or other venous graft |
|  | 38500-00 to 38500-04 | Coronary artery bypass, using 1 LIMA/RIMA/radial artery/epigastric artery/other artery graft |
|  | 38503-00 to 38503-04 | Coronary artery bypass, using >=2 LIMA/RIMA/radial artery/epigastric artery/other artery graft |
|  | 38505-00 | Open coronary endarterectomy |
|  | 38637-00 | Reoperation for reconstruction of coronary artery graft |
|  | 90201-00 to 90201-03 | Coronary artery bypass, using >=1 other graft, not elsewhere classified |
| 6^th^, 8^th^ | 38300-00, 38300-01 | Percutaneous or open transluminal balloon angioplasty of 1 coronary artery |
|  | 38303-00, 38303-01 | Percutaneous or open transluminal balloon angioplasty of >=2 coronary arteries |
|  | 38306-00 to 38306-05 | Percutaneous or open insertion of >=1 transluminal stent into single/multiple coronary artery |
|  | 38309-00, 38312-00, 38312-01,  38315-00,  38318-01 | Percutaneous transluminal coronary rotational atherectomy [PTCRA], >=1 artery with possible insertion of >=1 stent |
| 8^th^ | 38500-05, 38503-05 | Coronary artery bypass, using >=1 composite graft |
|  | 90218-00 to 90218-03 | Percutaneous transluminal coronary angioplasty with aspiration thrombectomy or embolic protection device, >=1 artery |
| **ICD-9-CM-A procedure codes** | |  |
|  | 3601, 3602 | Coronary artery (single vessel) angioplasty (laser) with/without thrombolytic agent infusion |
|  | 3603 | Open chest coronary artery angioplasty |
|  | 3605 | Coronary artery (multiple vessel) angioplasty (laser) |
|  | 3606 to 3607 | Insertion of non-drug or drug-eluting coronary artery stents(s) |
|  | 3609 | Other removal of coronary artery obstruction |
|  | 3610 | Aortocoronary bypass for heart revascularisation, not otherwise specified |
|  | 3611 to 3614 | Aortocoronary bypass of >=1 coronary artery |
|  | 3615 to 3616 | Single or double internal mammary-coronary bypass |
|  | 3619 | Other bypass anastomosis for heart revascularisation |
|  | 3699 | Other operations on vessels of heart |
