# Supplementary material for: Statin adherence is lower in primary than secondary prevention: A national follow-up study of new users
Source: PLoS One. 2020 Nov 19;15(11):e0242424. doi: 10.1371/journal.pone.0242424 (PMC7676659; doi:10.1371/journal.pone.0242424)
Supplement: S3 Table — (DOCX) [file pone.0242424.s003.docx]

**S3 Table : Medical Council of New Zealand (MCNZ) definitions**

| **Scope of practice** | **Definitions** |
| --- | --- |
| Provisional General Scope of practice | All new registrants, regardless of seniority, must work under supervision approved by MCNZ for at least 6 to 12 months to become familiar with New Zealand practice and culture.  During the supervised period, these registrants are registered within the Provisional General scope of practice and their performance is assessed by their supervisor approved by the MCNZ, in collaboration with senior colleagues. They will be required to complete certain requirements to be eligible for registration within the General scope. The only exception to this supervised period is for New Zealand and Australian graduates who have already completed their internship in Australia. |
| General Scope of practice | A doctor who has completed the requirements of a provisional general scope will be registered within a general scope of practice. Examples are doctors who have completed their first post-graduate year and may be in vocational training and doctors who have not started, or have chosen not to do, vocational training. |
| Provisional Vocational Scope of practice | A doctor who has completed their formal vocational training overseas, and whose qualifications, training and experience are considered either equivalent to, or as satisfactory as, that of a New Zealand-trained specialist may obtain Provisional Vocational registration. They must work under supervision for at least 6 to 18 months and complete our requirements for registration in a vocational scope. Those requirements may include undertaking some form(s) of assessment. |
| Vocational scope of practice | A doctor who has completed their vocational training as a specialist and has appropriate qualifications and experience can be registered within a vocational scope of practice. This form of registration recognises the doctor as a specialist and allows them to work independently in New Zealand. |
| Vocational: General Practice | General practice is an academic and scientific discipline with its own educational content, research, evidence base and clinical activity, and a clinical speciality orientated to primary care. It is personal, family, and community-orientated comprehensive primary care that includes diagnosis, continues over time and is anticipatory as well as responsive. |
| Vocational: Internal Medicine | Internal medicine involves the diagnosis and management of patients with complex medical problems which may include internal medicine, cardiology, clinical immunology, clinical pharmacology, endocrinology, gastroenterology, geriatric medicine, haematology, infectious diseases, medical oncology, nephrology, neurology, nuclear medicine, palliative medicine, respiratory medicine and rheumatology. |
| Vocational: Urgent Care | Urgent care medicine (formerly known as accident and medical practice) is the primary care of patients on an after-hours or non-appointment basis, where continuing medical care is not provided. |

**Source:** Medical Council of New Zealand (MCNZ) Glossary <https://www.mcnz.org.nz/about-us/glossary/> (accessed 28 August 2019)

MCNZ Types of vocational scope <https://www.mcnz.org.nz/registration/scopes-of-practice/vocational-and-provisional-vocational/types-of-vocational-scope/>

(accessed 28 August 2019)
